# Imagery ability of elite level athletes from individual vs. team and contact vs. no-contact sports

**DOI:** 10.7717/peerj.6940

**Published:** 2019-05-22

**Authors:** Donatella Di Corrado, Maria Guarnera, Francesca Vitali, Alessandro Quartiroli, Marinella Coco

**Affiliations:** 1 Department of Sport Sciences, Kore University, Enna, Italy; 2 Department of Psychology, Kore University, Enna, Italy; 3 Department of Neurosciences, Biomedicine, and Movement, University of Verona, Verona, Italy; 4 Department of Psychology, University of Wisconsin, Wisconsin, United States of America; 5 Department of Biomedical and Biotechnological Sciences, University of Catania, Catania, Italy

**Keywords:** Sport, Imagery vividness, Imagery controllability

## Abstract

**Background:**

In the sport context, imagery has been described as the condition in which persons imagine themselves while executing skills to deal with the upcoming task or enhance performance. Systematic reviews have shown that mental imagery improves performance in motor tasks

**Methods:**

The aim of the present study was to explore whether imagery vividness (i.e., the clarity or realism of the imagery experience) and controllability (i.e., the ease and accuracy with which an image can be manipulated mentally) differ by sport types (team vs. individual and contact vs. non-contact). Participants were athletes from team contact and non-contact sports (rugby and volleyball, respectively), and individual contact and non-contact sports (karate and tennis, respectively) between the ages of 20 and 33 years (*M* = 24.37, *SD* = 2.85). The participants completed the Vividness of Visual Imagery Questionnaire, the Vividness of Movement Imagery Questionnaire-2, and the Mental Image Transformation Tasks.

**Results:**

A 2 ×2 × 2 (gender × 2 contact-no-contact × 2 sport type) between groups MANOVA showed differences in imagery ability by sport type. Practical indications deriving from the findings of this study can help coaches and athletes to develop mental preparation programs using sport-specific imagery.

## Introduction

Mental imagery is a multisensory process that combines as many senses as possible to generate a vivid mental image. It plays a central role in the execution of movements and in human functioning. In the sport context, Watt, Spittle & Morris (2002) described imagery as the condition in which persons imagine themselves while performing skills. Visual (i.e., what an individual sees) and kinesthetic (i.e., sensory experience of the body while performing a movement) are the two most common sensory rehearsed mentally modes of generating images (Hall, 2001). Independently from the viewpoint (third or first person) or the mental mode (kinesthetic or visual), systematic reviews have shown that mental imagery improves performance in motor tasks and competitive situations, and facilitates motor acquisition and learning (Driskell, Copper & Moran, 1994; Cumming & Ramsey, 2009; Guillot & Collet, 2010; Moreau et al., 2010). Its advantages often depend on the ability to create vivid motor images; there seems to be a relationship between imagery ability (assemblage of skills including ease of image generation, image controllability, image vividness, and image conservation) and motor enhancement (Munroe et al., 2000). Research evidence suggests the use of different measures in the assessment of imagery ability (Williams et al., 2015; Guarnera et al., 2016; Cumming & Eaves, 2018). For example, researchers have investigated vividness (i.e., the clarity or realism of the imagery experience) indirectly via self-report questionnaires, which gauge the subjective perception of the quality of static and dynamic images. Imagery ability was also investigated measuring controllability, namely, the precision with which an image can be manipulated mentally (Roberts et al., 2008). Controllability can be measured through objective criteria such as the mental rotation, which requires cognitive manipulation, and spatial transformation of the imagined objects (Hall, Pongrac & Buckolz, 1985). In these experimental paradigms, participants are presented with stimuli (e.g., different 2-D letters or 3-D cubes) and they are asked to perform spatial manipulations of these stimuli to identify the correspondence of the items (Shepard & Metzler, 1971). Imagery was shown to play an important role in solving these tasks.

An individual can display high abilities in one type of imagery task, but less or none in others. Image vividness depends on the sensory modalities of the stimulus being imaged, capacity of cognitive processes, and individual differences (Bywaters, Andrade & Turpin, 2004). Controllability depends on differences in cognitive demands and neural pathways recruited (Carroll, 1993; Pearson et al., 2013; Castellano, Guarnera & Nuovo, 2015).

Several studies have investigated the frequency of imagery use in participants practicing different sports, using the Sport Imagery Questionnaire (SIQ; Hall et al., 1998). This instrument explores two cognitive factors: Cognitive Specific Imagery (movement and technique execution) and Cognitive General Imagery (tactics and action plans). The other three factors are Motivational Specific Imagery (winning, attaining successful performances and achieving goals), Motivational General-Arousal Imagery (emotional arousal related to competition in sport), and Motivational General-Mastery Imagery (control of emotions during challenging situations).

Across these areas of functioning, the effectiveness of imagery and the way that athletes employ it are influenced by different variables, such as gender, the type of skill (open vs. closed), competitive level (e.g., elite vs. non-elite performers), and sport types (team vs. individual sports; contact versus non-contact sports). Concerning gender, previous researches (Salmon, Hall & Haslam, 1994) found that men used imagery more consistently, and male athletes reported greater use of imagery than female athletes (Weinberg et al., 2003). Male athletes showed a greater ability to rotate mental images than female athletes (Schmidt et al., 2016). Moreover, a recent study found that men exhibited better spatial abilities than women (Habacha, Molinaro & Dosseville, 2014).

Open and closed skills differ in their relationship with the environment. In closed skills, it is easier to figure out the real movement that will be performed. On the contrary, open skills require the ability to imagine external events and changes to the environmental situations. Many individual sports involve closed skills, whereas many team sports involve open skills. Nevertheless, a clear classification of sports as typified by open or closed skills is difficult; indeed, many sports include a combination of both open and closed skills. In a study investigating imagery use by elite and novice athletes in open and closed sports, Arvinen-Barrow et al. (2007) found that open-skill athletes used significantly more Motivational general-arousal imagery than closed-skill athletes. Another research was conducted comparing 12 open-skills athletes, 12 closed-skills athletes, and 12 non-athletes in a mental rotation task (Ozel, Larue & Molinaro, 2004). Participants were required to mentally reorient, as quickly as possible, the test figure (on the right of the screen) by rotating it counterclockwise until the test figure was congruent with the standard figure. Results showed that to complete the mental rotation task, the non-athletes took 50% more time than the closed-skills and open-skills groups of athletes. The analysis did not show significant difference between the closed-skills and open-skills groups in mental rotation time. In line with these studies, Di Corrado, Guarnera & Quartiroli (2014) showed no difference in imagery ability (vividness and controllability) between open- and closed-skill sports, whereas dancers (closed skill) and karatekas (open skill) were found to report higher scores for imagery ability than the control group (non-athletes).

With respect to the expertise level, a recent study (Nezam et al., 2014) investigated vividness and ability of movement imagery in 256 elite, sub-elite and non-elite athletes (basketball, football, soccer, badminton, handball, and volleyball). Scores of internal visual and kinesthetic imagery were significantly higher in elite players than sub-elite and non-elite players. Furthermore, in a previous study Arvinen-Barrow et al. (2007) attempted to determine whether different-level athletes used the various imagery functions differently. They found that elite athletes used cognitive-related imagery more often than non-elite athletes.

Regarding the differences by sport types, researchers found that athletes playing team sports reported greater use of Motivational general–mastery imagery than athletes practicing individual sports, as found in soccer players (Short & Short, 2005; Adegbesan, 2009). Furthermore, other researchers (Short, Tenute & Feltz, 2005) found similar results with women volleyball, basketball, hockey, soccer, and softball players (team sports). In a previous study, Whitehead & Basson (2006) found that participants of an individual non-contact sport (squash) reported significantly less use of mental imagery (motivational general-mastery and cognitive general imagery) than karate (individual contact sport) and rugby (team contact sport) participants. In a recent study investigating the mental rotation performance, Moreau et al. (2011) found that elite combat athletes (i.e., fencing, judo, and wrestling) showed better mental rotation performance than elite runners.

Despite the large amount of imagery research in sport, very little attention has been devoted to the precise relationship between imagery ability and sport types. Given the limited research, the aim of the present study was to explore whether imagery vividness (i.e., the clarity or realism of the imagery experience) and controllability (i.e., the ease and accuracy with which an image can be manipulated mentally) differ by sport types (team vs. individual and contact vs. non-contact).

Further, we expected athletes of individual sports to show greater imagery ability than those of team sports. The hypothesis was based on the idea that successful performance often depends on the extent to which performers effectively identify, perceive, and use important sensory information (Hall, 2001). In individual sports, such as tennis and karate, the athletes do not interact with teammates as happens in team sports. The lack of interaction with teammates in individual sports likely involves a feeling of higher responsibility in the own actions, as well as lower environmental variability than team sports (Griffin, Mitchell & Oslin, 1997). These factors may stimulate the athletes of individual sports to form more specific mental representations of themselves while executing in comparison with athletes of team sports, and explain possible differences in imagery ability between individual and team sport performers.

## Materials & Methods

### Participants

The participants in this study were 120 injury-free national-level athletes (64 men and 56 women) from four different sports: tennis (16 men and 14 women), karate (16 men and 14 women), volleyball (14 men and 16 women), and rugby (18 men and 12 women). They ranged in age from 20 to 33 years (*M*_age_ = 24.37 years, *SD* = 2.85 years). All athletes had a minimum of 8 years of practice experience in the sport. They usually trained five or six times per week (*M* = 10.6 h, *SD* = 1.6 h). The sports chosen were representative of individual contact (karate), individual non-contact (tennis), team contact (rugby), and team non-contact (volleyball) sports.

The athletes were all members of sport clubs and were active competitors. Control participants were 60 university students (28 men and 32 women; *M*_age_ = 23.23 years, *SD* = 1.28 years) who had no experience in any sport. Participants did not receive previously mental skills or imagery education. Prior to the beginning of the study, ethical approval was granted from the first author’s university ethics committee. The study obtained ethical permission from the University Enna Kore Internal Review Board for psychological research (28 June 2018). All participants were informed about the procedures of the study and the anonymity of their answers before providing their written consent to participate, in accordance with the Declaration of Helsinki.

### Imagery assessments

#### The vividness of visual imagery questionnaire

The Vividness of Visual Imagery Questionnaire (VVIQ; Marks, 1973)) in the Italian adapted version (Antonietti & Crespi, 1995) was used to assess the participant’s imagery ability.

It is a 16-item (four items loading into four subscales) self-report instrument for evaluating how vividly individuals perform visual mental images (e.g., characteristics of a friend or parent, the climate, and the country). Once people have imagined a scene, they rate the images on clarity and vividness criteria on a five-point scale: 1: “*No image at all* (only “*knowing” that you are thinking of the object*)”; 2: “*Vague and dim*”; 3: “*Moderately clear and vivid*”; 4: “*Clear and reasonably vivid*”; and 5: “*Perfectly clear and vivid as normal*”. Higher scores indicate greater vividness (Antonietti & Crespi, 1995). Investigators reported a mean Cronbach’s *α* of .89 (McKelvie, 1995) and a 2-week test-retest reliability oscillating from *r* = .62 (Eton, Gilner & Munz, 1998) to *r* = .86 (Parrott & Strongman, 1985). Previous study (McKelvie, 1995) reported a criterion validity coefficient of *r* = .27 and concluded that there is evidence sustaining the Vividness of Visual Imagery Questionnaire as a valid measure.

#### The vividness of movement imagery questionnaire-2

The Vividness of Movement Imagery Questionnaire-2 (VMIQ-2; Roberts et al., 2008) is a self-report questionnaire for measuring imagery of movement and includes 12 items. Participants rate their ability to visually or kinesthetically imagine a movement using a 5-point Likert scale from 1 (*perfectly clear and vivid*) to 5 (*no image at all, you only know that you are “thinking” of the skill*). The VMIQ-2 measures three diverse kinds of imagery abilities: 1. External visual imagery (EVI), which denotes to an image obtained by observing movement performed by the subject from an outside perspective; 2. Internal visual imagery (IVI), that indicates an image obtained observing the subject him/herself while performing a movement; 3. Kinesthetic imagery (KIN), that describes the process of simulating the somatosensory experience of executing the task, as if individuals were really running. Previous study (Roberts et al., 2008) established the concurrent and construct validity of each factor of the VMIQ-2 with corresponding factors of the Movement Imagery Questionnaire—Revised (MIQ-R; Hall & Martin, 1997). The results showed that both the EVI and IVI factors of the VMIQ-2 were significantly correlated with the visual factor of the MIQ-R (*r* =  − .65, *p* < .01; *r* =  − .34, *p* < .05, respectively). The KIN factor of the VMIQ-2 was significantly correlated with the kinesthetic factor of the MIQ-R (*r* =  − .74, *p* < .01). The negative correlations are due to the two measures being scored in opposite directions.

#### Mental image transformation tasks

The validated Italian version of the Mental Imagery Test (MIT) was used (Di Nuovo, Castellano & Guarnera, 2014). The MIT includes eight tasks derived from different sources retrieved in mental imagery literature (Paivio, 1991; Campos, 2012), and designed to measure mental imagery skills, involving generation, maintenance, and manipulation of different categories of images. Di Nuovo and colleagues reported an *α* coefficient of *r* = .78 relative to the whole score of the Mental Imagery Test. For the current study, two of the tasks were administered.

#### Task cube

**Figure 1:**
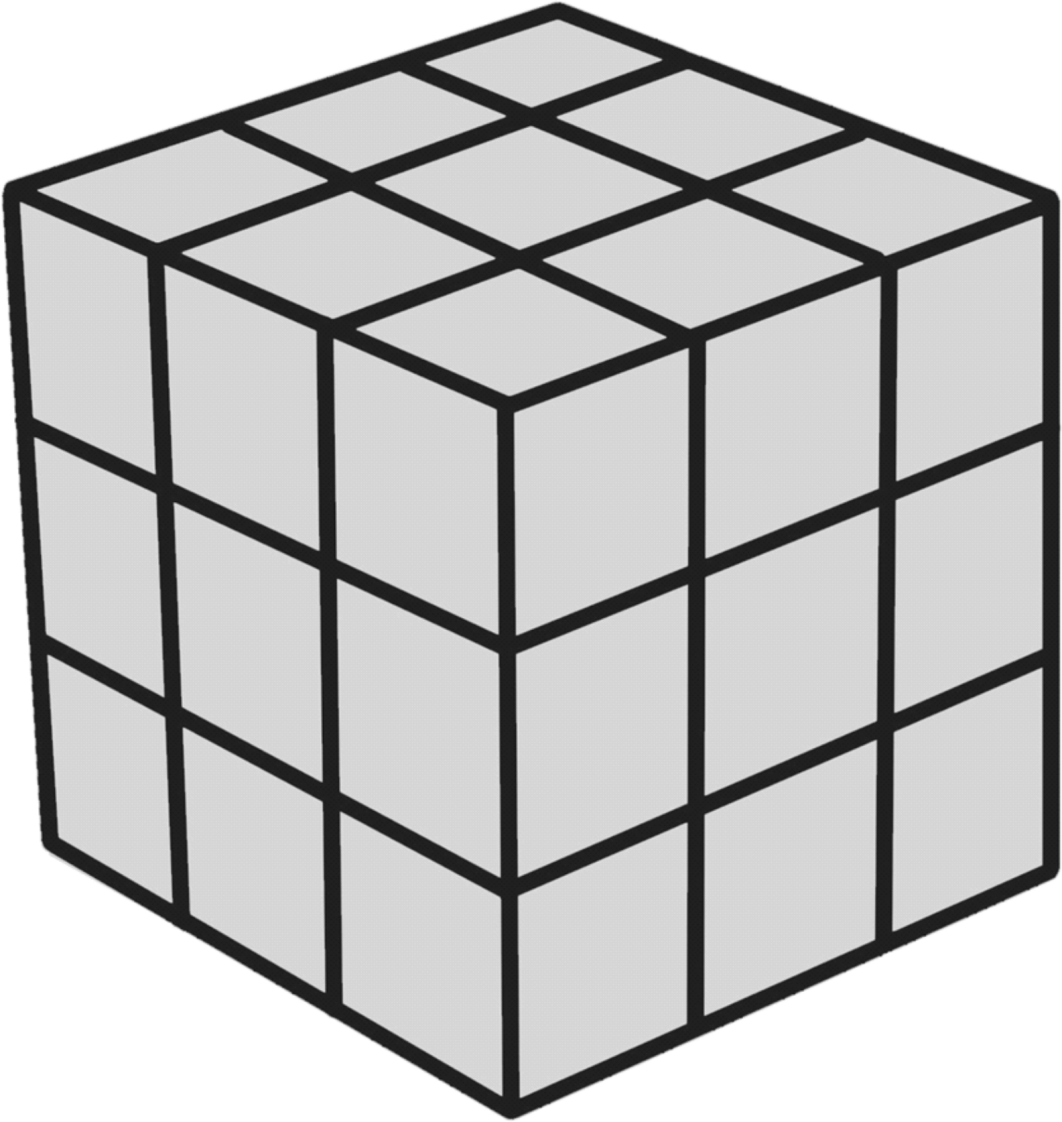
Mental image transformation task cube.

The picture of a large cube is shown for 30 s; it is composed of nine small cubes per face (3 × 3), and the external faces are coloured. After the stimulus is removed, the participant is asked to state how many small cubes have three external (coloured) faces, how many have two, how many one or none (Fig. 1).

#### Subtraction of parts

**Figure 2:**
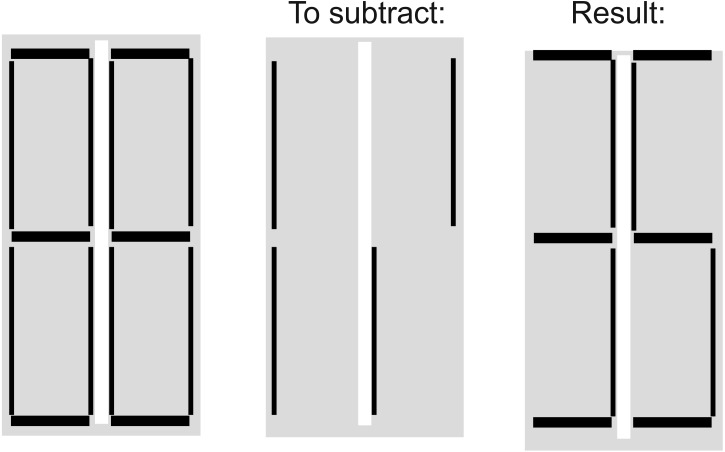
Mental image transformation task subtraction of parts.

A digital display with the number 88 composed of small segments is shown for 10 s. Then, another digital display with selected segments of a two-digit number is shown for 10 s. The participant is asked to imagine what two-digit number will remain after subtracting the parts of the new figure from the figure with all digits seen previously (Fig. 2).

### Procedure

The participants were examined separately, in a quiet room, in individual meetings lasting about 20 min. Measurements were conducted faraway the competitions to minimize any distractions. First, participants completed the vividness of the mental image questionnaires (the VVIQ and the VMIQ-2). For the image transformation task, we began by asking the participants to read the instructions on a computer screen. The participant was required to answer as quickly and as accurately as possible. During the test trials, no talking was allowed, and no feedback was provided. The control group was tested in a separate location near the university at the end of lectures.

### Data analysis

Statistical analyses were performed using the SPSS v. 25. A 2 ×2 × 2 (gender × contact/non-contact × individual/team) multivariate analysis of variance (MANOVA) was conducted on the data of the study variables. Follow up analysis of variance (ANOVA) and *t*-test with Bonferroni correction were then used to examine the locus of significant differences.

## Results

Findings revealed significant multivariate effects for individual vs. team sports, Wilks *λ* = .837, *F* (6, 107) = 3.482, *p* = .003, ${\eta }_{\mathrm{p}}^{2}=.163$, power = .937, and on the interaction between individual vs. team sports and contact vs. non-contact sport, Wilks *λ* = .710, *F*(6, 107) = 7.294, *p* = .001, ${\eta }_{\mathrm{p}}^{2}=.290$, power = 1.000. Means and standard deviations of study variables are reported in Table 1.

Follow up ANOVAs revealed significant univariate differences between individual and team sports on VVIQ and EVI variable scores. Specifically, athletes of individual sports reported higher scores on Vividness of Visual Imagery ability, *F*(1, 112) = 6.160, *p* = .015, ${\eta }_{\mathrm{p}}^{2}=.052$, power = .692 (individual *M* = 59.30; team *M* = 54.40), and on External Visual Imagery, *F*(1, 112) = 6.237, *p* = .014, ${\eta }_{\mathrm{p}}^{2}=.053$, power = .697 (individual *M* = 25.02; team *M* = 29.18) than athletes of team sports.

**Figure 3:**
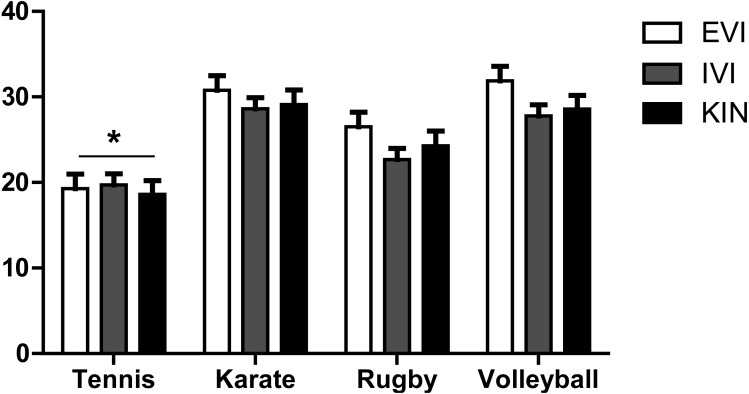
Variable scores by sport. EVI, External Visual Imagery, IVI, Internal Visual Imagery, KIN, Kinesthetic Imagery. *, *p* < .001. Error bars indicate standard error of the mean.

Post hoc *t*-tests confirmed that athletes of individual non-contact sport showed greater vividness for motor imagery combining kinesthetic and visual properties compared with athletes of the other sports. Specifically, significant differences emerged in the interaction between individual/team × contact/non-contact sports regarding the following abilities (Fig. 3):

**Table 1:** Mean ± SD for the study variables. Each data point indicates the mean and standard deviation for the variables.

		Men				Women			
		Contact		No contact		Contact		No contact	
Variables		Indiv *n* = 16	Team *n* = 18	Indiv *n* = 13	Team *n* = 14	Indiv *n* = 14	Team *n* = 12	Indiv *n* = 17	Team *n* = 16
VVIQ									
	M	56.06	63.28	60.15	48.50	57.86	49.25	62.88	53.44
	SD	9.03	12.75	9.98	14.35	7.93	17.80	6.91	16.40
EVI									
	M	29.50	26.44	19.77	36.50	32.21	26.58	18.88	27.81
	SD	10.25	10.78	5.37	8.03	9.66	10.71	4.79	11.44
IVI									
	M	27.50	23.11	20.62	30.64	29.93	22.00	19.00	25.31
	SD	7.92	7.93	4.79	7.56	6.99	6.52	4.06	7.52
KIN									
	M	28.00	23.83	20.08	33.36	30.36	25.00	17.47	24.37
	SD	11.55	10.92	5.22	8.96	7.72	7.55	4.86	11.11
Clock									
	M	6.63	7.50	6.38	5.86	5.64	7.17	6.12	6.94
	SD	1.20	.78	.87	2.62	2.50	.72	1.17	1.98
Cube									
	M	3.13	3.33	3.54	4.14	3.86	2.67	4.47	3.25
	SD	2.72	3.31	1.66	3.37	2.53	2.99	3.04	3.41

• External Visual Imagery, *F*(1, 112) = 25.537, *p* < .001, ${\eta }_{\mathrm{p}}^{2}=.186$, power = .999. In the tennis group, significantly higher scores were observed on the variable EVI (tennis vs. karate *p* < .001; tennis vs. rugby *p* < .001; tennis vs. volleyball *p* < .001).
• Internal Visual Imagery, *F*(1, 112) = 30.416, *p* < .001, ${\eta }_{\mathrm{p}}^{2}=.214$, power = 1.000. In the tennis group, significantly higher scores were found on the variable IVI (tennis vs. karate *p* < .001; tennis vs. volleyball *p* < .001).
• Kinesthetic Imagery, *F*(1, 112)= 20.175, *p* < .001, ${\eta }_{\mathrm{p}}^{2}=.153$, power = .994. In the tennis group, higher scores were shown on the variable KIN (tennis vs. karate *p* < .001; tennis vs. volleyball *p* < .001; tennis vs. rugby *p* = .006).

Scores on the Mental Image Transformation Tasks did not yield significant differences among groups. Moreover, there was no significant difference (*p* > .05) across groups concerning gender.

## Discussion

The aim of the present study was to explore whether imagery vividness (i.e., the clarity or realism of the imagery experience) and controllability (i.e., the ease and accuracy with which an image can be manipulated mentally) differ by sport types (team vs. individual and contact vs. non-contact). These results demonstrate that individual and team athletes have distinct abilities and characteristics when it comes to sport-oriented imagery. Mainly, the statistical analysis supported the hypothesis that athletes practicing individual sports exhibit better imagery ability than those of team sports. Specifically, in the tennis group, significantly higher scores were observed on the variables External Visual Imagery, Internal Visual Imagery and Kinesthetic Imagery. This result was not found in previous studies.

Sport type also affects the use of visual perspective, which can be either internal or external depending on the sport (Hall, 2001; Di Corrado et al., 2015). External visual imagery was found more effective for form-based tasks as athletes could effortlessly imagine the global movements and positions that are essential for successful performance (Hardy & Callow, 1999). Internal visual imagery would be higher in goal-directed tasks or motor skills that include fast changes in the visual field (Callow & Roberts, 2012). Conversely, kinesthetic imagery involved the perceptions of how it feels to perform, including the strength and energy supposed during movement (Callow & Waters, 2005). For example, tennis players require an internal visual imagery while feeling arm movements and effort needed for serving. In contrast, an external visual imagery is required to visualize the ball trajectory and its rebound after serve. Regarding the imagery use, Weinberg et al. (2003) reported that athletes practicing individual sports use more motivation general arousal type of imagery compared to athletes playing team sports. Fogarty & Morris (2003), examining imagery-perspective use, found that junior elite tennis performers tended to use more internal than external imagery.

In partial confirmation of such findings, Hale et al. (2005) suggested that it is easier, through internal visual imagination, to feel the perception of movement, therefore internal visual imagination seems to be related with kinesthetic imagination. In this regard, Hall, Rodgers & Barr (1990) claimed that internal vision has the potential to integrate kinesthetic imagery, while external vision is not enough to create such sensations. No other studies have been published to support these claims of systematic differences in imagery ability.

Furthermore, kinesthetic imagery ability was found to be higher for tennis athletes. This may be related to the nature of tennis, where the necessity of kinesthetic ability is higher in the actual performance, and in which the movement depends on environmental signals such as the adversary’s body language and feedforward approximations of ball motion or opponent movement. Moreover, in individual sports, such as tennis and karate, the lack of interaction with teammates involves a feeling of higher responsibility in the own actions, as well as lower environmental variability than team sports (Griffin, Mitchell & Oslin, 1997). These factors may stimulate the athletes of individual sports to form more specific mental representations of themselves while executing in comparison with athletes of team sports, and explain possible differences in imagery ability between individual and team sport performers.

In our study, there was no significant difference across groups concerning gender. The results are in line with previous research showing mixed results in imagery ability by gender (Munroe et al., 2000; Hall, 2001; Whitehead & Basson, 2006). Moreover, we did not find any significant differences in Mental Image Transformation Tasks between groups; one reason for this set of results may be that the involved athletes not required mental rotation or transformation skills in order to perform successfully. This is consistent with a study by Jansen & Lehmann (2013) who compared three groups (soccer players, gymnasts, and non-athletes) in an object-based mental rotation task consisting of human postures and cube figures. They found that the gymnasts showed a better mental rotation performance than non-athletes.

In conclusion, results of the present research showed differences in imagery ability across sports.

Findings of this study highlight the need for coaches and athletes to recognize the specific requirements of their sport to effectively incorporate mental imagery for performance enhancement in their preparation routines.

## Conclusions

While the different nature (individual vs. team-based; contact vs. non-contact) of the sports considered is a strength of this study, athletes from only four sports were involved due to the difficulty to recruit athletes of similar competitive level and ability. This incomplete representation of sport types is a limitation and, therefore, future studies on imagery ability should recruit a larger number of athletes from a wider variety of sports.

Moreover, this study did not consider the potential causal relationships between the practice of different types of sports and imagery vividness and imagery controllability. Future research should be conducted using longitudinal analysis or experimental protocols to assess possible causal relationships. From an applied perspective, the findings of this study can suggest practical indications for coaches and athletes to effectively develop mental preparation programs using sport-specific imagery.

##  Supplemental Information

10.7717/peerj.6940/supp-1 Supplemental Information 1 Raw Data Raw data set of the Vividness of Visual Imagery Questionnaire (VVIQ), External Visual Imagery (EVI), Internal Visual Imagery (IVI), and Kinesthetic Imagery (KI).

## Additional Information and Declarations

### Data Availability

The following information was supplied regarding data availability: The raw data are available as a Supplemental File.
